# Mediation analysis of gestational age, congenital heart defects, and infant birth-weight

**DOI:** 10.1186/1756-0500-7-926

**Published:** 2014-12-17

**Authors:** Adane F Wogu, Christopher A Loffredo, Ionut Bebu, George Luta

**Affiliations:** Department of Biostatistics, School of Public Health, University of North Carolina at Chapel Hill, Chapel Hill, NC USA; Departments of Oncology and of Biostatistics, Bioinformatics, and Biomathematics, Georgetown University, 3800 Reservoir Rd, NW, Washington, DC 20057 USA; Department of Epidemiology and Biostatistics, The Biostatistics Center, The George Washington University, Rockville, USA

**Keywords:** Birth weight, Congenital heart defects, Gestational age

## Abstract

**Background:**

In this study we assessed the mediation role of the gestational age on the effect of the infant’s congenital heart defects (CHD) on birth-weight.

**Methods:**

We used secondary data from the Baltimore-Washington Infant Study (1981–1989). Mediation analysis was employed to investigate whether gestational age acted as a mediator of the association between CHD and reduced birth-weight. We estimated the mediated effect, the mediation proportion, and their corresponding 95% confidence intervals (CI) using several methods.

**Results:**

There were 3362 CHD cases and 3564 controls in the dataset with mean birth-weight of 3071 (SD = 729) and 3353 (SD = 603) grams, respectively; the mean gestational age was 38.9 (SD = 2.7) and 39.6 (SD = 2.2) weeks, respectively. After adjusting for covariates, the estimated mediated effect by gestational age was 113.5 grams (95% CI, 92.4–134.2) and the mediation proportion was 40.7% (95% CI, 34.7%–46.6%), using the bootstrap approach.

**Conclusions:**

Gestational age may account for about 41% of the overall effect of heart defects on reduced infant birth-weight. Improved prenatal care and other public health efforts that promote full term delivery, particularly targeting high-risk families and mothers known to be carrying a fetus with CHD, may therefore be expected to improve the birth-weight of these infants and their long term health.

## Background

Congenital heart defects (CHD), also known as congenital cardiovascular malformations, are the most common type of structural birth defects and have substantial effects on pediatric mortality and morbidity [1, 2]. The prevalence of CHD in the live-born population of the United States is estimated to be 1 out of 125 births [3, 4], and approximately 40,000 infants are born annually with CHD [5]. In addition, the 2009 annual summary of vital statistics in the United States showed that CHDs were the leading cause of all birth defect-related infant deaths [6].

CHDs originate in the early weeks of pregnancy when the heart is forming and are usually diagnosed either prenatally or soon after birth, although some defects are not diagnosed until later in childhood or even in adulthood. Among the most consistently observed findings in newborns with CHD are reduced birth weight [3, 7–11] and increased incidence of clinically low and very low birth weight infants, i.e. <2,500 g and <1,500 g, respectively [3, 10, 12, 13]. An additional highly consistent finding is that infants with CHD are often born prematurely, suggesting that the association of CHD with low birth weight may be attributed, at least in part, to prematurity or reduced gestational age [8, 14–18]. Whether CHD, low birth weight, and prematurity are all co-outcomes of a common, underlying teratogenic influence such as maternal environmental exposure is plausible but as yet unknown [19–21].

The aim of our study was to investigate the relationship between the CHD status of infants and their birth weight by considering gestational age as a mediating variable (mediator), i.e., a variable that “mediates” the relation between the predictor (an infant’s CHD status) and the outcome (birth weight). Using statistical mediation analysis, we considered gestational age as the mediator, we assessed its indirect effect on the relationship of interest, and we estimated the proportion of the effect of CHD status on infant birth weight accounted for by this mediator, taking into account infant and maternal covariates known to affect birth weight and CHD status.

## Methods

### Study population

The Baltimore-Washington Infant Study was a population-based case–control study of the potential risk factors of CHD, conducted from 1981 to 1989 in Maryland, the District of Columbia, and Northern Virginia. The study enrolled all live-born infants with CHD and a randomly selected sample of controls that was representative of all regional live births. Controls were randomly sampled at birth hospitals in the region during the same time period. Parents of cases and controls were interviewed at home by trained personnel within one year of the infant’s birth and asked questions concerning medical and demographic/socioeconomic factors, as well as questions regarding cigarette smoking and alcohol consumption. Mothers reported the infant’s birth weight in pounds and ounces, which we converted to grams for this analysis. Gestational age was calculated from the mother’s report of the infant’s date of birth in relation to her reported expected delivery date [1, 13]. The diagnosis of each case was made during the first year of life and confirmed by pediatric cardiologists using echocardiography, cardiac catheterization, surgery, or autopsy. Detailed information about the Baltimore-Washington Infant Study dataset as well as the study in general has been reported previously [1, 22, 23]. The study was approved by the Institutional Review Board of the University of Maryland, Baltimore.

From the extensive Baltimore-Washington Infant Study dataset, we selected variables that are known from prior reports to influence both infants’ CHD status and birth weight, as potential confounders for our study. These included an infant’s sex [24–26] and race [27–29], as well as maternal age [27, 30, 31], education [31], pre-existing maternal diabetes [32, 33], parity [30, 34, 35], and cigarette smoking during the first trimester [19, 31, 32, 35]. We excluded twins and other multiple births due to their much smaller birth weights relative to singletons and their high prevalence of premature births.

### Statistical analysis

In mediation analysis, the main interest is to assess how much the effect of an exposure on a response variable is mediated through an intermediate variable, oftentimes called the mediator [36]. Thus, the intermediate variable lies on the causal pathway between the exposure (independent variable) and the response (dependent variable). Based on the statistical mediation approach proposed by Baron and Kenny [37], the mediation analysis is carried out by partitioning the total effect of the independent variable on the dependent variable into its mediated (or indirect) effect and its direct effect [38]. The mediated effect is the effect of the independent variable on the dependent variable accounted for by the mediator, whereas the direct effect is the effect of the independent variable on the dependent variable holding the mediator constant. Imai et al. [39] and MacKinnon [40] operationalized the procedure using a regression-based approach by proposing the implementation of three separate, but related, regression models. In the first model (Model 1), the dependent variable is regressed on the independent variable; in the second model (Model 2), the mediator is regressed on the independent variable; and in the third model (Model 3), the dependent variable is regressed on both the mediator and the independent variable. An attractive feature of this regression-based approach is that additional potential confounders and other terms (e.g., interactions between covariates) can be included.

After obtaining parameter estimates from these three regression models, we estimated the mediated effect as the product of the estimate of the effect of the independent variable on the mediator from Model 2 with the estimate of the effect of the mediator on the dependent variable from Model 3 [37, 40–43]. The 95% confidence intervals for the mediation effect were calculated and compared using three different approaches: (1) the Wald statistic approach, based on the Sobel test [44]; (2) the likelihood ratio test approach [40], which is based directly on the likelihood function rather than on the quadratic approximation of the log-likelihood function used by the Wald statistic approach; and (3) the bootstrap approach [43, 45–47].

From an epidemiological point of view, in order to explore the mechanism of exposure-disease relations, it is important to assess the mediated (indirect) effect. A relevant way of expressing mediated effects is by considering the “proportion mediated”, which is the proportion of the total effect explained by a particular mediator [38–40, 48]. The mediation proportion was estimated by taking the ratio of the estimate of the mediated effect to the estimate of the total effect (i.e., the effect from Model 1). We used and compared three different approaches [40] to calculate the 95% confidence intervals for the mediation proportion: (1) the Delta method [49] to compute the standard error of the mediation proportion so as to construct the confidence intervals for the mediation proportion; (2) the Fieller’s method [50]; and (3) the bootstrap approach [45, 51–53]. The results from the bootstrap approach were compared, for the purpose of justification, with the results from the other two approaches. During the bootstrap approach, we used 500 draws (i.e., bootstrap resamples) for point estimation as well as construction of confidence intervals. The mediation analyses were performed using SAS 9.3 (SAS Institute Inc., Cary, NC) and R (R Foundation for Statistical Computing, Vienna, Austria).

### Ethical standards

The parents of all participants signed an informed consent agreement to participate in the original Baltimore-Washington Infant Study protocol, which was approved by the Instututional Review Board of the University of Maryland, Baltimore. The present analysis was performed on data abstracted without identifiers.

## Results

The Baltimore-Washington Infant Study enrolled a total of 3,377 cases and 3,572 controls from 1981 to 1989. Fifteen cases and 8 controls were dropped from the analyses due to missing values. Characteristics of the study population are shown in Table 1. The average birth weights of the cases and controls were 3071 (Standard deviation = 729) and 3353 (Standard deviation = 603) grams, respectively (*p* < 0.0001), confirming the expected deficit among the infants with CHD. The average gestational ages were found to be 38.9 (standard deviation = 2.7) and 39.6 (standard deviation = 2.2) weeks, respectively, and the difference was statistically significant (*p* < 0.0001). Infant sex, maternal education, and maternal cigarette smoking were distributed similarly in cases and controls (p > 0.05), whereas the case–control differences in infant race, maternal age, previous pregnancies, pre-pregnancy diabetes, and method of delivery were each statistically significant. Based on these results we explored these variables as potential covariates in the subsequent statistical models.

**Table 1:** **Characteristics of infants in the BWIS dataset**

	Group		p-value
	Cases ^1^ (n = 3362)	Controls (n = 3564)	
**Dependent and mediating variables**			
Birth–weight, grams: mean (SD)	3071 (729)	3353 (603)	<0.0001
Gestational age, weeks: mean (SD)	38.9 (2.7)	39.6 (2.2)	<0.0001
**Covariates**			
**Infant’s sex (%)**			
male	1661 (49.41)	1812 (50.84)	0.2320
female	1701 (50.59)	1752 (49.16)	
**Infant’s race (%)**			
white	2175 (64.69)	2358 (66.16)	
African American	1057 (31.44)	1108 (31.09)	0.0274
other	130 (3.87)	98 (2.75)	
**Mother’s age at conception (SD) years**	26.72 (5.92)	26.27 (5.72)	0.0014
**Previous pregnancies (%)**			
never pregnant	1014 (30.16)	1154 (32.38)	0.0006
one	986 (29.33)	1154 (30.72)	
two	661 (19.66)	709 (19.89)	
≥ three	701 (20.85)	606 (17.00)	
**Maternal smoking status (%)**			
Non smoker	2150 (63.95)	2298 (64.48)	0.3363
1–10 cigarettes/day	535 (15.91)	565 (15.85)	
11–20 cigarettes/day	480 (14.28)	526 (14.76)	
21–39 cigarettes/day	143 (4.25)	136 (3.82)	
40 or more cigarettes/day	54 (1.61)	39 (1.09)	
**Mother’s education (%)**			
6 years, or less, completion	26 (0.78)	22 (0.62)	
7–9 years completion	220 (6.57)	228 (6.42)	
10–11 years completion	358 (10.7)	405 (11.4)	0.2248
12 years completion	1265 (37.8)	1259 (35.42)	
13 years, or more, completion	1478 (44.16)	1640 (46.15)	
**Pre-pregnancy diabetes (%)**			
No	3283 (98.09)	3531 (99.35)	<0.0001
Yes	64 (1.91)	23 (0.65)	
**Method of Delivery**			
Vaginal	2307 (68.34)	2694 (75.42)	<0.0001
Caesarian section	1069 (31.66)	878 (24.58)	

^1^Cases included 123 with laterality and looping defects, 578 with conotruncal (outflow tract) defects, 288 with endocardial cushion (atrioventricular septal) defects), 275 with pulmonic valve stenosis, 519 with left-sided obstructive defects, 859 with ventricular septal defect, 290 with atrial septal (secundum) defect, 82 with patent arterial duct, and 347 with other cardiovascular malformations.

The parameter estimates, standard errors, and *p-*values from the three regression models (Model 1, Model 2, and Model 3) are shown in Table 2. During the model building steps, interaction terms between the variables were considered, but none of them were found to be statistically significant (*p* > 0.10; where p = 0.10 was used as statistical cut-point for the inclusion of interaction terms into the final models). Race of the infant, maternal age, education and parity were not statistically significant in any of the models considered and were not included in the final models. Two covariates – sex of the infant and maternal smoking status – were associated with infant birth weight, with or without gestational age in the model. In Model 1, the CHD status had a statistically significant detrimental effect on birth weight (*p* < 0.0001, Table 2(a)). Adjusted for sex of infants and maternal smoking status, on average, infants with CHD had a birth weight 279 g lower (95% confidence interval, 248–310) than that of infants without CHD. The CHD status also had a small but statistically significant association with gestational age in Model 2 (*p* < 0.0001, Table 2(b)). When CHD status and gestational age of infants were both considered as predictors of the birth weight of the infants (Model 3), both variables were found to be statistically significant (*p* < 0.0001 each, Table 2(c)). Adjusted for gender, maternal smoking, and gestational age, the difference between the birth weight of infants with CHD and infants without CHD decreased to 165.9 g (95% confidence interval, 141–190).

**Table 2:** **Results from the three regression models: having birth weight as the dependent variable (Model 1 and Model 3) and having gestational age as the dependent variable (Model 2)**

Variable	Parameter estimate	Standard error	t–value	p–value
(a) **Model 1** (dependent variable is birth weight in grams)				
Intercept	3349.39	14.82	226.07	<0.0001
CHD^1^	−279.00	15.82	−17.64	<0.0001
Child’s sex^2^	137.53	15.81	8.70	<0.0001
1–10 cigarettes/day^3^	−191.44	22.14	−8.64	<0.0001
11–20 cigarettes/day^3^	−167.19	22.96	−7.28	<0.0001
21–39 cigarettes/day^3^	−224.75	40.60	−5.54	<0.0001
40 or more cigarettes/day^3^	−240.76	68.92	−3.49	0.0005
(b) **Model 2** (dependent variable is gestational age in weeks)				
Intercept	39.64	0.06	708.67	<0.0001
CHD^1^	−0.69	0.06	−11.54	<0.0001
Child’s sex^2^	0.04	0.06	0.64	0.5198
1–10 cigarettes/day^3^	−0.26	0.08	−3.16	0.0016
11–20 cigarettes/day^3^	−0.21	0.09	−2.46	0.0138
21–39 cigarettes/day^3^	−0.21	0.15	−1.38	0.1662
40 or more cigarettes/day^3^	−0.07	0.26	−0.27	0.7880
(c) **Model 3** (dependent variable is birth weight in grams)				
Intercept	−3157.30	99.75	−31.65	<0.0001
Gestational age	164.13	2.50	65.68	<0.0001
CHD^1^	−165.92	12.53	−13.24	<0.0001
Child’s sex^2^	131.23	12.41	10.57	<0.0001
1–10 cigarettes/day^3^	−148.04	17.39	−8.51	<0.0001
11–20 cigarettes/day^3^	−132.14	18.03	−7.33	<0.0001
21–39 cigarettes/day^3^	−189.91	31.87	−5.96	<0.0001
40 or more cigarettes/day^3^	−229.28	54.09	−4.24	<0.0001

^1^Reference is infants without CHD.

^2^Reference is female infants group.

^3^Reference is nonsmoker mothers group.

### Analysis of the mediated effect and mediation proportion

From these three regression models, we observed that the total effect of CHD status on infant birth weight was 279 g (Table 2(a)). The direct effect of the CHD status on birth weight was 166 g (Table 2(c)), and the mediated effect was 164.13 × 0.69 = 113 g (95% confidence interval, 93.6–132.6 based on the Wald statistic approach, and 99.9–117.8 based on the likelihood-based approach; Table 3(a)). The point estimate of the mediated effect found from the bootstrap approach was 113.5 g, which is closely comparable to the estimates found from Wald statistic and likelihood-based approaches (95% confidence interval, 92.4–134.2, Table 3(a)). The 95% confidence interval from the likelihood-based approach was narrower compared to the Wald statistic and bootstrap approaches.

**Table 3:** **Point estimates and 95% confidence intervals (CI) of the mediated effect and mediation proportion using three approaches**

**(a). MEDIATED EFFECT**			
**Estimation method**	**Wald statistic method**	**Likelihood-based method**	**Bootstrap method**
**Estimate (95% CI)**	113.08 (93.6–132.6)	113.08 (99.9–117.8)	113.51 (92.4–134.2)
**(b). MEDIATION PROPORTION**			
**Estimation method**	**The delta method**	**Fieller’s method**	**Bootstrap method**
**Estimate (95% CI)**	40.5% (32.2%–48.8%)	40.5% (35.2%–45.9%)	40.7% (34.7%–46.6%)

The estimated mediation proportion was 40.5% using the Wald statistic (Delta) and Fieller’s methods (95% confidence intervals, 32.2%–48.8% and 35.2%–45.9%, respectively), and 40.7% (95% confidence interval, 34.7%–46.6%) using the bootstrap method (Table 3(b)). In other words, adjusted for gender and maternal smoking, approximately 41% of the effect of the CHD status on birth weight was mediated by the gestational age of the infant, and the remaining 59% was a direct effect of CHD status on birth weight.

We also investigated the mediation proportion for the two sexes separately. The mediation proportion was 45.2% (95% Fieller’s confidence interval, 36.6%–54.0%) for males and 36.7% (95% Fieller’s confidence interval, 29.8%–43.5%) for females; the difference in these two proportions was not statistically significant (*p* = 0.3342).

## Discussion

In studying the association between an independent and dependent variable, there are situations in which the independent variable affects the outcome variable both directly and indirectly, by influencing a third variable (the mediator) which in turn influences the dependent variable. Thus, the relationships between variables are often complex and cannot be well modeled by a single regression equation, while consideration of a mediator allows researchers to more fully explore the separate and combined effects of interest. The problem of CHD as a potential cause of reduced birth weight in affected infants is amenable to this approach because birth weight itself is highly dependent on gestational age, which in turn may be influenced by CHD to some extent. Consistent with this view, several studies have previously documented the association of CHD in newborns with their decreased birth weight and gestational age, relative to unaffected infants, but to our knowledge, the mediation effect has not been directly assessed in prior published research [1, 13]. Additionally, we were able to account for the effects of additional covariates of birth weight in our models: males had consistently higher birth weights than females on average in this study, while maternal smoking resulted in decreased birth weight in a dose-dependent manner. Both effects were independent of gestational age. Caesarian section, a possible indicator for a planned inducement of early delivery for a fetus known or suspected to have birth defects, was more common among cases than controls. While it reduced the mean gestational age of cases by 0.5 weeks (*p* = 0.002) and had a lesser affect on controls (−0.3 weeks, *p* = 0.99), the use of caesarian section did not significantly affect the mean birth weights of infants in this study.

This study has several strengths and limitations. The data is from the Baltimore-Washington Infant Study, which had achieved complete ascertainment of cases in the region and a population-based random sample of controls; therefore selection bias is extremely unlikely as an explanation for our findings. On the other hand, we cannot rule out the possibility of recall bias of infant birth weight, nor random (non-systematic) errors in recalling the weight or the estimated date of delivery. There are also statistical issues that should be considered in evaluating our results. We confined our analysis to the linear effects of variables on birth weight, but future work should consider possible non-linear effects as well. While our use of regression models permitted us to assess these mediation effects, the technique can become unwieldy for more complex situations [40, 44, 54]. For example, the original Baltimore-Washington Infant Study dataset contains a large number of variables that could be explored jointly as mediators and/or covariates. Furthermore, it would be interesting to consider a common teratogen model, in which a factor such as maternal smoking was modeled as affecting both the occurrence of the malformation and contributing simultaneously to premature birth and decreased weight at birth.

## Conclusions

In summary, we used data from a population-based epidemiological study to evaluate the mediation effect of gestational age in explaining the relationship between CHD and decreased birth weight of infants. The results suggest that this mediation accounts for a substantial proportion (about 41%) of the latter association, which was not apparent in previous research in this field due to the limitations of conventional statistical association methods. Further research is needed to confirm these findings in other populations and to explore even more complex methods such as structural equation modeling [55, 56].
